# Tissue Cytometry With Machine Learning in Kidney: From Small Specimens to Big Data

**DOI:** 10.3389/fphys.2022.832457

**Published:** 2022-03-04

**Authors:** Tarek M. El-Achkar, Seth Winfree, Niloy Talukder, Daria Barwinska, Michael J. Ferkowicz, Mohammad Al Hasan

**Affiliations:** ^1^Division of Nephrology, Department of Medicine, Indiana University, Indianapolis, IN, United States; ^2^Department of Pathology and Microbiology, University of Nebraska Omaha, Omaha, NE, United States; ^3^Department of Computer and Information Science, Indiana University–Purdue University Indianapolis, Indianapolis, IN, United States

**Keywords:** 3D imaging, cytometry analysis, kidney injury, artificial intelligence, deep learning

## Abstract

Advances in cellular and molecular interrogation of kidney tissue have ushered a new era of understanding the pathogenesis of kidney disease and potentially identifying molecular targets for therapeutic intervention. Classifying cells *in situ* and identifying subtypes and states induced by injury is a foundational task in this context. High resolution Imaging-based approaches such as large-scale fluorescence 3D imaging offer significant advantages because they allow preservation of tissue architecture and provide a definition of the spatial context of each cell. We recently described the Volumetric Tissue Exploration and Analysis cytometry tool which enables an interactive analysis, quantitation and semiautomated classification of labeled cells in 3D image volumes. We also established and demonstrated an imaging-based classification using deep learning of cells in intact tissue using 3D nuclear staining with 4′,6-diamidino-2-phenylindole (DAPI). In this mini-review, we will discuss recent advancements in analyzing 3D imaging of kidney tissue, and how combining machine learning with cytometry is a powerful approach to leverage the depth of content provided by high resolution imaging into a highly informative analytical output. Therefore, imaging a small tissue specimen will yield big scale data that will enable cell classification in a spatial context and provide novel insights on pathological changes induced by kidney disease.

## Introduction

Understanding the biology and function of an organ requires detailed assessment of various cells and structures in the intact tissue environment (Asp et al., 2019; Stewart et al., 2019; Barwinska et al., 2021). This is particularly needed for the kidney, an organ with complex architecture where zonation of specialized cells and structures is directly linked with physiological function (Hato et al., 2013; El-Achkar and Dagher, 2015; Berry et al., 2017; Barwinska et al., 2021; Ferkowicz et al., 2021). Furthermore, disease states are associated with alteration in tissue architecture and changes in cell distribution, activity, and/or state (Wilson et al., 2019; Lake et al., 2021; Muto et al., 2021). Technological advancements such as single cell RNA sequencing that provide high content information at the cell and molecular levels have enhanced our ability to further classify cells into subtypes, and study alterations in cell states, which could be linked to disease pathogenesis and outcomes (Park et al., 2018; Lake et al., 2019, 2021; Wilson et al., 2019; Menon et al., 2020). Innovative approaches in high content and high-volume imaging of kidney tissue are also rapidly evolving (Winfree et al., 2017a,^2018^, ^2021^; Singh et al., 2019; Black et al., 2021a,b; Ferkowicz et al., 2021; Lipp et al., 2021; Liu et al., 2021; Melo Ferreira et al., 2021; Neumann et al., 2021), and these advancements are urgently needed to: (1) provide a platform of discovery based on imaging data, thereby delivering a unique context within an intact tissue environment and (2) anchor and interpret *in situ* emerging findings from technologies that lose the spatial context (Winfree et al., 2021). In the last decade, we saw an evolution of imaging kidney tissue from a qualitative toward a highly quantitative science (Winfree et al., 2017b,^2021^; Singh et al., 2019; Martins et al., 2020; Black et al., 2021a,b; Melo Ferreira et al., 2021; Neumann et al., 2021). This progress has been enhanced by the advancements in various modalities of microscopy that could perform high-resolution large-scale imaging. The ability to image multiple labels simultaneously (multiplexing) has significantly increased the depth of content acquired (Singh et al., 2019; Woloshuk et al., 2020; Ferkowicz et al., 2021; Melo Ferreira et al., 2021; Neumann et al., 2021). Furthermore, imaging in all 3 dimensions using optical sectioning has allowed faithful preservation of tissue architecture and spatial context (Puelles et al., 2016; Klingberg et al., 2017; Winfree et al., 2017b; Ferkowicz et al., 2021; Lake et al., 2021; Liu et al., 2021). These advancements were catalyzed by the availability of novel software tools that allow streamlined image processing and quantitative analysis (Dao et al., 2016; Winfree et al., 2017a; Czech et al., 2019; Stoltzfus et al., 2020). These significant developments were discussed during the 2021 Indiana University O’Brien Center for Advanced Microscopy Analysis workshop (Dunn et al., 2021).

**FIGURE 1:**
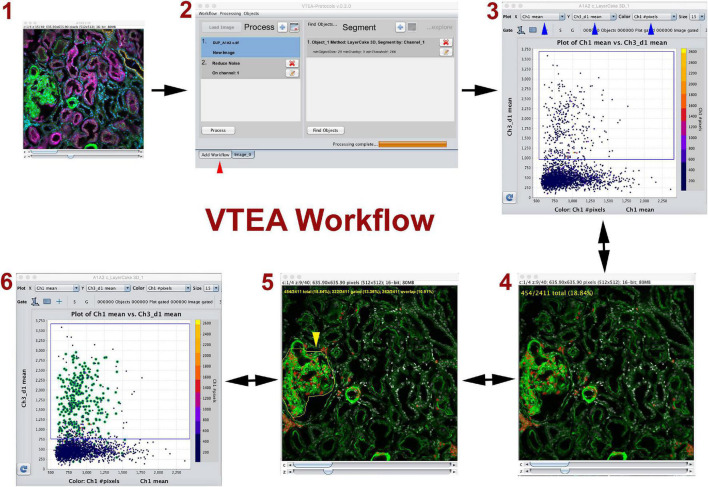
Volumetric Tissue Exploration and Analysis (VTEA) basic workflow. VTEA is a user-friendly platform that allows interactive exploration of image volume (1) where image processing, segmentation, analysis and exploration could occur in a single workspace (2). In the analytical plot, each dot represents a cell with various features. The simplest analysis is in the form of a 2D scatter plot displaying features on the *x* and *y* axis (3). Gates can be drawn to chose and quantify a specific population of cells that can be directly visualized in the image volume (4). Conversely, regions of interest can be drawn in the image (5) to locate cells of interest in the scatter plot (6). This process allows for an explorative interplay between the image and the analytical space. Red arrowhead shows different tabs available in the workspace. Figure adapted and used with permission from Winfree et al. (2017b).

In this mini-review we will focus on advancement in large scale 3D imaging of kidney tissue and analysis using tissue cytometry with the Volumetric Tissue Exploration and Analysis (VTEA) software tool (Figure 1; Winfree et al., 2017b; Ferkowicz et al., 2021). We will also discuss how incorporating novel machine learning approaches and algorithms with tissue cytometry has enhanced the ability to expand and transform the analysis of image volumes toward discovery (Winfree et al., 2021). Particularly, developing deep neural networks that allow classification of cells independent of specific labels will not only increase the power and usefulness of cytometry in classifying cells based on imaging data (Woloshuk et al., 2020), but will also enable unbiased and non-exhaustive discovery of cell subtypes *in situ*. These novel subtypes can then be visualized and mapped back in the image volumes, which will allow biological interpretation. Therefore, this could become a unique opportunity whereby the learning could become interpretable. Furthermore, when large scale 3D imaging is coupled with advanced computational tools that allow processing of large image volumes, hundreds thousand cells or more could be analyzed from a single tissue specimen, thereby allowing the generation of big data from these imaging experiments.

## Tissue Cytometry

Tissue cytometry refers to the process of surveying all cells within an image volume of a tissue, and transforming cells into “analysis-ready” objects with associated variables based on labels (such as fluorescence marker intensities) or spatial parameters. Frequently, the nuclei are used as fiduciaries for the cells because: (1) nuclear staining can be easily incorporated into most experimental designs, and (2) nuclei can be consistently segmented using several standard approaches (Winfree et al., 2017b,^2021^; Dunn et al., 2019). The segmented nuclei representing individual cells could then be used in an analytical pipeline that allows quantitative analysis based on the various parameters associated with each cell. The simplest form of analysis is a plot displaying 2 dimensions in the *x* and *y* axis, where specific gates could be drawn based on a threshold such as fluorescent label intensity (Figure 1). Two key components of tissue cytometry are obtaining quantitative measurements of the cell populations of interest *and* direct visualization by mapping back the cells of interest into the image volume. The latter allows on-the-spot validation of the “choice” of cells (whether by direct gating or other methods) and biological interpretation (particularly when specific distribution patterns start to emerge). Multiple software tools (open-source or commercial) have been developed to perform image analysis, and can be used for tissue cytometry (Gerner et al., 2012; Winfree et al., 2017a; Stoltzfus et al., 2020, 2021; Stirling et al., 2021). We have described the VTEA tool (Winfree et al., 2017b), which was applied specifically to perform tissue cytometry on 3D image volumes of kidney tissue (Figure 1). Potential advantages of VTEA include: open-source as a plugin to ImageJ, a single platform that allows image processing, segmentation and cytometry analysis, extensibility and easy incorporation of novel computational approaches, leveraging existing ImageJ tools for image analysis, interactive interplay between the image volume and the analytical process used. We have used tissue cytometry with VTEA in various settings such as to study the abundance and distribution of epithelial and immune cells in the mouse and human kidney (Winfree et al., 2017b; Ferkowicz et al., 2021; Lake et al., 2021), understand the association of epithelial and immune cells to injury in the setting of human acute and chronic kidney disease and stone disease (Lake et al., 2021), quantify and localize the activation of c-JUN in the mouse kidney (Lafavers et al., 2019), study changes in lymphatics in various models of kidney injury (Black et al., 2021a,b). Large scale 3D imaging and tissue cytometry with VTEA is a key tissue interrogation technology used by the Kidney Precision Medicine Project (KPMP) consortium to extract cellular and molecular information from kidney biopsies of patients with kidney disease (De Boer et al., 2021; El-Achkar et al., 2021; Lake et al., 2021). Therefore, the application of tissue cytometry in analyzing kidney tissue is expanding, and has proven to be complementary to other technologies that do not preserve the tissue architecture.

## Tissue Cytometry and Machine Learning

Since multiple parameters can be extracted for each single cell using high resolution multiplexed imaging, advancing the analytical approach to take into account the effect of all these parameters in thousands of cells becomes a big data problem. It is then reasonable to incorporate machine learning algorithms to help cluster, classify and visualize cell subtypes into the analytical space. Indeed, the extensibility of VTEA to incorporate available libraries of machine learning algorithms is a significant development that enables a semi-automated unsupervised classification of cells (Winfree et al., 2022). We demonstrated that this approach could be useful in classifying cells from reference kidney tissue (Woloshuk et al., 2020). In addition, the ability to understand cell-cell and cell-structure interactions could be enhanced by performing neighborhood analysis, as implemented, for example in CytoMAP or VTEA (Stoltzfus et al., 2020; Lake et al., 2021; Winfree et al., 2022). We recently used VTEA to perform a cell centric neighborhood analysis on >1.2 million cells from various kidney biopsies of patients with kidney disease (Lake et al., 2021). This approach uncovered spatial associations that were validated by other transcriptomics-based technologies. One of the key advantages of performing such cell-centric neighborhood analysis (percent of cells within a distance from each cell) is the ability to merge analysis from various specimens into one analytical space, since such analysis is by default normalized (Lake et al., 2021).

**FIGURE 2:**
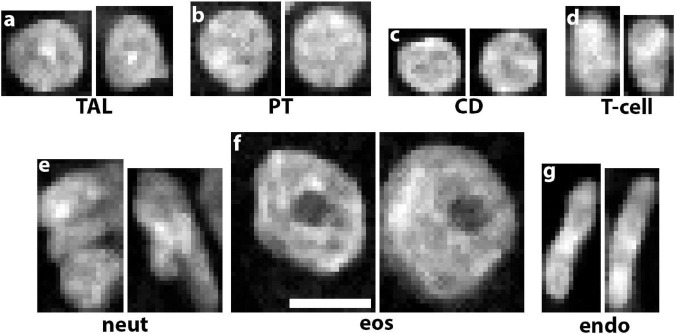
Unique nuclear staining signatures of various kidney cell types. DAPI staining alone reveals distinct signatures of chromatin condensation states and nuclear morphology of **(a)** thick ascending limbs (TAL), **(b)** proximal tubules (PT), **(c)** collecting ducts (CD), **(d)** T-cells, **(e)** neutrophils, **(f)** eosinophils, and **(g)** endothelial cells. Scale bar = 5 μm.

Multiplexing various probes into one imaging experiment offers significant advantages for cell classification based on particular labels. For example, using highly multiplexed detection such as imaging mass cytometry or co-detection by indexing allows the classification of multiple cell subtypes (Singh et al., 2019; Melo Ferreira et al., 2021; Neumann et al., 2021). However, multiplexing has limitations, particularly in its application in 3D and its practicality when kidney tissue is of limited availability (Winfree et al., 2021). In addition, using pre-specified labels limits the potential of agnostic discovery of novel cell types and subtypes based on imaging data. To circumvent these limitations, we recently devised a deep learning approach to classify cells based only on nuclear staining (Woloshuk et al., 2020). The premise is based on the fact that nuclear staining has unique features for each cell type (Figure 2) and its changes could represent alterations in cell states (Gustafsdottir et al., 2013; Eulenberg et al., 2017). Therefore, these studies are confined within a biologically interpretable context (Woloshuk et al., 2020). This work presented us also with a unique opportunity to test several unexplored questions such as: is 2D enough or do we need the information in 3D image volumes of nuclei? Can we use classical machine learning classifiers that extract features or do a deep neural network work better? Does the context of the nucleus (i.e., neighboring nuclei) improve cell classification accuracy? Our results showed that we could successfully classify cells from human reference kidneys into eight different classes based on machine learning approaches, but the highest accuracy was achieved with a 3D deep neural network trained on 3D image volume of nuclei with context (Woloshuk et al., 2020). Our efforts are currently to extend this approach to kidney disease, and use the 3D leaning network to uncover cell subtypes induced by injury. This could be done by using various approaches. For example, the features extracted by the 3D network from the nuclear staining could be used to reclassify and visualize cells using tissue cytometry. Importantly, novel machine learning tools could be applied on these features to achieve non-exhaustive learning and agnostically discover new cell subtypes that can be further vetted using tissue cytometry. This will be discussed next.

## Leveraging Machine Learning for Agnostic Discovery

**FIGURE 3:**
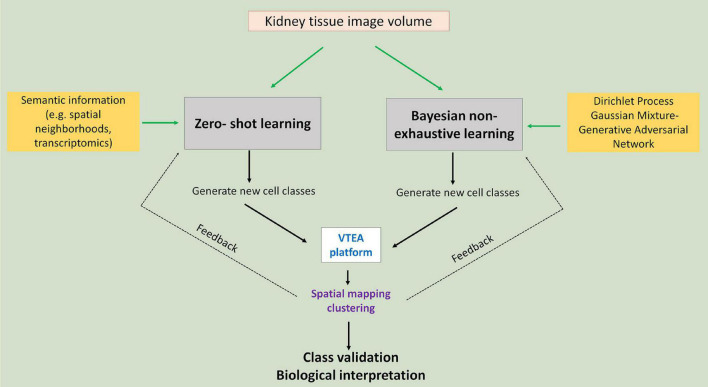
Agnostic discovery using machine learning and tissue cytometry. Proposed approach to use imaging data of cell nuclei in machine learning workflows that allow non-exhaustive classification of new classes that could be visualized and further analyzed using tissue cytometry. Green arrows point to the two proposed approaches: zero-shot and Bayesian non-exhaustive learning.

Agnostic discovery is the exploration process for the identification and localization of novel kidney cell subtypes induced by injury. In an agnostic discovery scenario, obtaining labeled cell examples for the injury cell subtypes is a hard task for many reasons: first, the nature of injury to the morphology of kidney cells due to disease is unknown so accurate labeling is difficult; second, we may not yet have a suitable marker for such cells, which makes us unable to correctly label them using cytometry; finally, due to lack of knowledge regarding the injury it is even hard for us to know the definite count of number of possible injury subtypes. While lack of labeled data makes agnostic discovery a difficult task, recent advances in supervised classification can help us in this regard (Figure 3).

In supervised classification, identifying novel classes (example: novel injury states) for which no examples are available in the training data (ground truth datasets used in training machine learning classifier) has received wide-spread attention from the machine learning community in recent years. There are different approaches for solving such machine learning tasks. Most prominent among these is called zero-shot learning (ZSL), which is well studied by the deep learning community (Romera-Paredes and Torr, 2015; Zhang and Saligrama, 2015). ZSL is also becoming a promising direction in the medical domain. In recent works, ZSL has been used in diagnosis and classification of disease in chest radiographs (Hayat et al., 2021; Paul et al., 2021). Bayesian non-exhaustive classification is another prominent direction (Görür and Edward Rasmussen, 2010; Ben-Yosef and Weinshall, 2018).

For zero-shot learning, the number of novel classes along with side-information (also known as semantic information) about all the classes needs to be provided upfront. During training, the learning algorithm utilizes the side information to compensate for the lack of labeled data for the unknown class. Spatial neighborhood data around a cell can be used as side-information. For instance, we expect that the concentration of inflammatory immune cells (such as neutrophils or T-cells) around injured kidney cells would be higher, and hence, such side information will be relevant for classifying injured kidney cell subclass instances. Another potential candidate for side information is to use data from alternate modality, say, single cell RNA sequencing and/or spatial transcriptomics data. Using such data will not only help us identify novel kidney cells, but also will provide more information regarding the pathways that control the injury progression in the kidney cells over time. A challenge of zero-shot learning is that it requires that the number of novel classes is known during training time, which is often not feasible for agnostic discovery. In that case, Bayesian non-exhaustive classification can be used. Using Bayesian technique, it learns some parametric probability distribution for the known classes. During inference, it identifies instances which are far away from the distribution of the known classes and create a new class along with its probability distribution. Generally, Dirichlet process Gaussian Mixture Model is used for non-exhaustive classification (Görür and Edward Rasmussen, 2010; Zhuang and Al Hasan, 2021). The challenge in Bayesian non-exhaustive classification is that their performance becomes very poor if the assumed data distribution does not follow the actual data distribution.

## Challenges for Image-Based Classification in Human Kidney Biopsies

The novel imaging-based approaches discussed to characterize cell types and subtypes in human kidney tissue specimens are very promising. However, it is also important to discuss some of the challenges and limitations that need to be addressed to make these methodologies more robust and accessible. First, variation in tissue processing practices and fixation may alter the quality of the tissue and the downstream imaging data. The effects of changes in tissue processing on the ability to classify cells using tissue cytometry and machine learning are unknown. Fortunately, collaborative studies (such as the KPMP consortium) that are focused on interrogating kidney tissue biopsy specimens are rigorously standardizing tissue acquisition and processing, which would allow to set standards and perform comparison with data acquired from archived tissues originating from other sources (De Boer et al., 2021; El-Achkar et al., 2021). Second, it is possible that some of the changes in cell states that are induced by disease may not be accompanied by significant alteration in nuclear activity or morphology. Therefore, expanding classification strategies to include another common marker that tracks changes in cell morphology and activity in the cytoplasm such as F-actin will likely increase the sensitivity and dynamic range of capturing subtle changes in cell states. Finally, performing imaging and data analysis is frequently limited to centers with appropriate expertise and resources, which may limit accessibility to the broader research community. Furthermore, the computational breadth needed for data access, storage and transfer may also be a restrictive factor. Therefore, increasing the accessibility of these approaches by using and disseminating open-source software, public imaging data repositories and accessible cloud-based imaging visualization and analysis tools will provide reasonable first steps to make these innovative tools more reachable by the broader community.

## Conclusion and Future Outlook

We highlighted in this mini-review advances in tissue cytometry of kidney tissue, emphasizing novel analytical approaches that transform imaging-based data into highly quantifiable big data outputs that can also be used for discovery while incorporating the richness of the spatial context. These advances are crucial to understand kidney disease, which frequently displays regional heterogeneity at the cellular and molecular levels. Leveraging novel machine learning approaches will allow unbiased discoveries such as novel cell types and subtypes which are spatially anchored and linked to other features that allow biological interpretation. In the future, we anticipate that with relatively few labels, the combination of tissue cytometry with machine learning will enable a form of enhanced “*virtual multiplexing*,” which could classify most cell types *in situ* within kidney tissue and allow the agnostic discovery of novel cell types based on imaging. For the kidney, imaging and analyzing tissue will certainly become a very important issue!

## Author Contributions

All authors listed have made a substantial, direct, and intellectual contribution to the work, and approved it for publication.

## Conflict of Interest

The authors declare that the research was conducted in the absence of any commercial or financial relationships that could be construed as a potential conflict of interest.

## Publisher’s Note

All claims expressed in this article are solely those of the authors and do not necessarily represent those of their affiliated organizations, or those of the publisher, the editors and the reviewers. Any product that may be evaluated in this article, or claim that may be made by its manufacturer, is not guaranteed or endorsed by the publisher.
